# Anti-cyclic citrullinated peptide antibody in bronchoalveolar lavage fluid of patients with idiopathic pulmonary fibrosis

**DOI:** 10.1186/s12890-025-04088-9

**Published:** 2026-01-08

**Authors:** Ryobu Mori, Kohei Ikezoe, Tomohiro Handa, Kiminobu Tanizawa, Kazuko Uno, Toyohiro Hirai

**Affiliations:** 1 Department of Respiratory Medicine, Graduate School of Medicine, Kyoto University, 54 Shogoin Kawahara-cho, Sakyo-ku, Kyoto, 606-8507 Japan; 2 Department of Advanced Medicine for Respiratory Failure, Graduate School of Medicine, 54 Shogoin Kawahara-cho, Sakyo-ku, Kyoto, 606-8507 Japan; 3 Division of Respiratory Medicine, Center for Respiratory Diseases, National Hospital Organization Kyoto Medical Center, 1-1 Fukakusa-Mukaihata-cho, Fushimi-ku, Kyoto, 612-0861 Japan; 4 Division of Basic Research, Louis Pasteur Center for Medical Research, 103-5 Tanaka- Monzen-cho, Sakyo-ku, Kyoto, 606-8225 Japan

**Keywords:** Anti-cyclic citrullinated peptide antibody, Idiopathic pulmonary fibrosis, Bronchoalveolar lavage fluid, Rheumatoid arthritis, Biomarker

## Abstract

**Background:**

Idiopathic pulmonary fibrosis (IPF) and rheumatoid arthritis-associated interstitial lung disease (RA-ILD) share several risk factors, genetic backgrounds, and morphological features, including the usual interstitial pneumonia pattern on computed tomography and histopathology. Anti-cyclic citrullinated peptide antibodies (ACPAs) are RA-related autoantibodies that are associated with RA-ILD. ACPA production can be induced in the lungs of patients with IPF.

**Methods:**

Forty-four patients with IPF and 10 patients with RA-ILD underwent bronchoalveolar lavage fluid (BALF) collection. The concentrations of IgG ACPAs in the BALF were measured and corrected for total IgG levels. The relationships between corrected BALF ACPA levels and clinical features were investigated.

**Results:**

The proportion of ACPAs (ACPA-IgG level adjusted by total IgG level) in the BALF was significantly lower in patients with IPF than in those with RA-ILD (1222 ± 1424 U/mg vs. 9058 ± 15159 U/mg, *p* < 0.01). Compared with the group with low ACPA proportions (*n* = 29), the IPF group with high ACPA proportions (*n* = 15) in the BALF was younger (65.2 ± 9.4 years vs. 70.9 ± 6.8 years, *p* = 0.03) and had more females (6 out of 15 (40%) vs. 2 out of 29 (7%), *p* = 0.01). Additionally, the IPF patients with a high ACPA proportion in the BALF had significantly better outcomes than those with a low ACPA proportion did (median overall survival time: 92.4 months vs. 41.1 months, *p* = 0.04).

**Conclusion:**

The corrected BALF ACPA level might be an important biomarker for identifying IPF phenotypes with favourable outcomes.

**Supplementary Information:**

The online version contains supplementary material available at 10.1186/s12890-025-04088-9.

## Introduction

Idiopathic pulmonary fibrosis (IPF) is a chronic, progressive fibrosing interstitial pneumonia with a poor prognosis and a median survival time of 3–5 years if left untreated [1, 2]. IPF is characterized by recurrent alveolar epithelial injury followed by abnormal fibroblast activation and excessive extracellular matrix deposition. Recent studies suggest that both intrinsic factors, such as genetic predisposition, and extrinsic factors, including environmental exposures, contribute to disease progression through multiple molecular signaling pathways [3, 4]. However, despite these advances in understanding IPF pathogenesis, only two antifibrotic drugs are currently approved. Moreover, the natural history of IPF is highly variable and unpredictable, and current antifibrotic therapies can only slow disease progression without halting or curing the disease. Therefore, there remains an unmet need for biomarkers that can predict disease progression and outcomes, elucidate disease mechanisms, and thereby help identify novel therapeutic targets.

Rheumatoid arthritis (RA) is a common autoimmune disease that can cause interstitial lung disease (ILD). ILD affects 10 to 60% of patients with RA, depending on the definition used and the study population; importantly, the incidence of ILD is associated with shortened survival [5–11]. IPF and RA-associated ILD (RA-ILD) share several risk factors and genetic components [12], such as older age, male sex, smoking [13, 14] and MUC5B promoter variant polymorphisms [15, 16], . Moreover, RA-ILD frequently shows the usual interstitial pneumonia (UIP) pattern on high-resolution computed tomography (HRCT) and histopathology [7, 11, 17], which is a specific morphological feature of IPF. Thus, there may be mechanisms shared between IPF and RA-ILD [10].

Anti-cyclic citrullinated peptide antibodies (ACPAs) are RA-related autoantibodies, and ACPA positivity is associated with ILD in RA patients, with a specificity of 95–98% [18–20]. Exposure to foreign agents, including cigarette smoke or agents causing respiratory infection, can induce citrullination of proteins where activated peptidylarginine deiminase replaces arginine with citrulline and leads to ACPA production in susceptible individuals. Thus, the lung might be the initial site of ACPA production [21]. Furthermore, the citrullination pathway has been reported to be upregulated in IPF patients [22–24].

In the present study, we hypothesised that ACPAs may be highly produced in the lungs of a substantial proportion of IPF patients and that such patients have specific clinical features. The aims of our study were to measure ACPA levels in bronchoalveolar lavage fluid (BALF) and identify the relationships between ACPA levels in the BALF and clinical features, including quantitative CT indices, and outcomes in patients with IPF.

## Methods

### Patient selection

All patients who underwent BALF collection at our institution from September 2008 to February 2018 were screened. Patients who were diagnosed with IPF according to the ATS/ERS/JRS/ALAT guidelines for IPF published in 2022 [25] were included. Patients who experienced acute exacerbation or had active malignant disease were excluded. Ten patients with RA-ILD were also included as disease controls.

### Data collection

Clinical, physiological, and laboratory data were retrieved from medical records. The baseline characteristics included age, sex, BMI, smoking history (pack-years), and laboratory test results, including lactate dehydrogenase (LDH), C-reactive protein (CRP), Krebs von den Lungen-6 (KL-6), surfactant protein D (SP-D), and serum ACPA levels. BALF collection, arterial blood gas analysis while breathing room air, pulmonary function tests, six-minute walk tests, and HRCT were also performed during the process of diagnosis. For the pulmonary function test, the percent-predicted forced vital capacity (FVC), forced expiratory volume in 1 s over forced vital capacity (FEV_1_/FVC), and percent-predicted diffusing capacity of the lung for carbon monoxide (%DLCO) were measured [26, 27]. The modified Gender, Age, and lung Physiology (GAP) index score was calculated, and staging was performed as previously reported [28]. The six-minute walk test was performed in accordance with the ATS guidelines [29]. Treatments provided after BALF collection, survival, and causes of death were also recorded.

### Bronchoalveolar lavage fluid collection

The affected lung segments as noted on the chest CT images were targeted, and BALF collection was performed by instilling 50 mL of sterile normal saline into the lung segments through a flexible bronchoscope and retrieving the fluid. The procedure was repeated three times. The BALF was pooled, filtered through sterile gauze to remove mucus strands and centrifuged at 1500 rpm for 10 min at 4 °C, and the supernatant was stored at − 80 °C until the analysis of ACPA and total IgG levels.

### Measurement of IgG-type ACPAs, total IgG antibodies, and IgA-type ACPAs in the BALF

The concentrations of IgG ACPA and total IgG in the BALF were measured via ELISA. IMMUNOSCAN CCPlus^®^ (RA-96PLUSRUO, Svar Life Science, Sweden) was used for the measurement of IgG ACPAs (U/mL) in the BALF, and total IgG (ng/mL) was measured via an IgG (Total) Human Uncoated ELISA Kit with plates (88-50550-22, Thermo Fisher Scientific, USA). Both types of antibodies were measured according to the manufacturers’ instructions, and the analytical performance was verified by assessing linearity, detection capability, and precision. The ACPA concentration in the BALF of each patient was normalised to the total IgG concentration for the same patient, and the normalised value was called the ACPA proportion (U/mg). Additionally, IgA ACPAs in the BALF were qualitatively assessed using an Anti-CCP ELISA kit (MyBioSource, USA) in a subset of patients (*n* = 34), due to the limited availability of archived BALF samples.

### Measurement of serum anti-cyclic citrullinated peptide antibodies

The serum ACPA concentration was measured with an ELISA kit (MESACUP-2 test CCP; MBL Inc., Japan; the cut-off value was set at 4.5 U/mL) until March 2017 and was measured with a chemiluminescent immunoassay using ARCHITECT (Abbott Japan, Japan; the cut-off value was set at 4.5 U/mL) after April 2017. The results are described as positive or negative on the basis of the cut-off value.

### Artificial intelligence-based quantitative CT analysis

Artificial intelligence-based quantitative CT image analysis software (AIQCT) was applied to HRCT images at the time of BAL examination. AIQCT automatically detects and quantifies bronchi, vessels and eight types of parenchymal patterns (normal lungs, ground‒glass opacity, reticulation, consolidation, honeycombing, nodules, interlobular septum, and hyperlucency) and provides the volume and percentage of each feature [30]. In addition to the eight types of parenchymal patterns, the fibrosis volume was calculated as the volumetric sum of the reticulation and honeycombing, and the ILD volume was calculated as the volumetric sum of the ground‒glass opacities and fibrosis.

### Statistical analysis

Continuous variables are presented as the means and standard deviations (SDs). Student’s t test was used for comparisons of continuous variables, whereas the chi-square test or Fisher’s exact test was used for comparisons of categorical variables, as appropriate. The Spearman’s rank correlation coefficient was calculated to evaluate the association between two variables that were not normally distributed. The Kaplan‒Meier method was used to estimate all-cause mortality. The log-rank test was used to compare overall survival between different groups. The Cox proportional hazards model was used to assess the associations between clinical variables and survival time. All the statistical analyses were performed via EZR version 1.55 (Saitama Medical Centre, Jichi Medical University, Saitama, Japan). For all analyses, *p* < 0.05 was considered statistically significant.

## Results

### ACPA concentrations in the BALF of IPF and RA-ILD patients

**Fig. 1:**
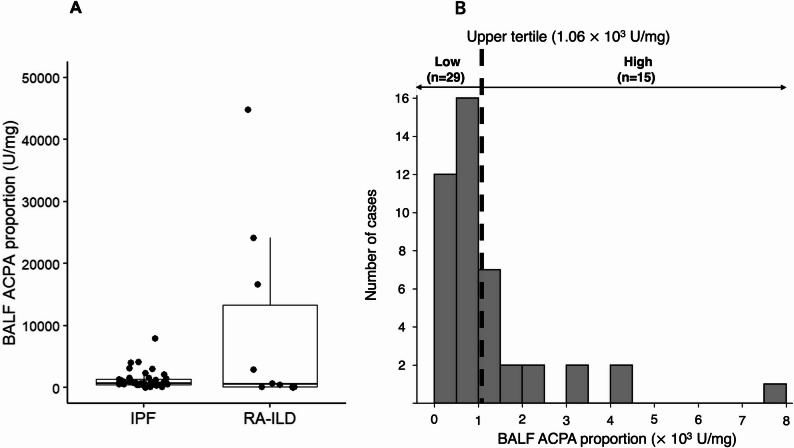
Comparison and distribution of BALF ACPA proportion in IPF and RA-ILD **A** Comparison of the anti-cyclic citrullinated peptide antibody (ACPA) proportion (ACPA level/total IgG level) in bronchoalveolar lavage fluid (BALF) between patients with idiopathic pulmonary fibrosis (IPF) (n=44) and those with rheumatoid arthritis-associated interstitial lung disease (RA-ILD) (n=10) **B** Histogram showing distribution of BALF ACPA proportion (ACPA level/total IgG level) in patients with IPF. The histogram bins use inclusive lower bounds and exclusive upper bounds. The dotted line indicates the upper tertile (cutoff = 1.06 ⨯ 10^3^U/mg), used to define high (*n*=15) and low (*n*=29) ACPA proportions

The levels of IgG ACPAs in the BALF of 44 patients with IPF and 10 patients with RA-ILD were measured, and the ACPA proportions, the ACPA values corrected according to the total IgG level in the BALF, were compared. The mean proportion of ACPAs in the BALF was significantly lower in IPF patients than in RA-ILD patients (1222 ± 1424 U/mg vs. 9058 ± 15159 U/mg, *p* < 0.01; Fig. 1A). Figure 1B shows that the ACPA proportion in BALF from IPF patients was not normally distributed (*p* < 0.01, the Shapiro-Wilk test) and a certain number of patients with IPF had a high ACPA proportion; therefore, these patients were divided into two groups according to the upper tertile of the ACPA proportion as the cut-off value: patients with a high ACPA proportion (*n* = 15) and those with a low ACPA proportion (*n* = 29).

### Characteristics of IPF patients with high/low ACPA proportions

The baseline characteristics of the study subjects, IPF patients with high and low proportions of ACPAs in the BALF and patients with RA-ILD, are shown in Table 1. Among the 44 patients with IPF, surgical lung biopsy was performed in 8 patients. No patients had been treated with either antifibrotics or immunosuppressants, including glucocorticoids, at the time of BALF collection. Compared with those with low ACPA proportions, IPF patients with high ACPA proportions were significantly younger (65.2 ± 9.4 years vs. 70.9 ± 6.8 years, *p* = 0.03) and more likely to be women (6 out of 15 (40%) vs. 2 out of 29 (7%), *p* = 0.01). The serum ACPA level was positive in only one patient whose ACPA proportion was high but was negative in all other patients. There were no significant differences between the two groups regarding the serum KL-6 level, pulmonary function test (PFT) data, GAP stage, or six-minute walk test results. In terms of the BALF results, the total cell count and cell fraction did not significantly differ between the two groups.

**Table 1:** Baseline characteristics of the study participants

	IPF High ACPA proportion (*n* = 15)	IPF Low ACPA proportion (*n* = 29)	*p* value	RA-ILD (*n* = 10)
Demographic data				
Age	65.2 (9.4)	70.9 (6.8)	0.03	65.3 (12.4)
Sex M/F	9/6	27/2	0.01	5/5
BMI (kg/m^2^)	24.4 (4.4)	25.1 (3.6)	0.56	24.0 (2.7)
Smoking status Current / Past / Never	1/11/3	2/25/2	0.42	2/4/4
Pack-years	50.9 (46.1)	39.1 (24.2)	0.27	22.9 (25.9)
WBC (/mm^3^)	6443 (2066)	6943 (1186)	0.31	8280 (2580)
LDH (U/L)	213 (43)	225 (37)	0.36	200 (45)
CRP (mg/dL)	0.19 (0.25)	0.33 (0.51)	0.32	4.07 (6.29)
KL-6 (U/mL)	933 (640)	1010 (535)	0.67	808 (874)
SP-D (ng/mL)	245 (321)	282 (170)	0.62	109 (113), *n* = 3
Serum ACPA +/–	1/14	0/29	0.34	8/2
Pulmonary function testing				
%FVC	84.5 (22.2)	74.3 (19.2)	0.12	NA
FEV_1_/FVC	82.7 (10.5)	85.3 (6.3)	0.31	NA
%DLCO	53.7 (14.3)	52.7 (18.1)	0.85	NA
GAP stage (I/II/III)	10/5/0	10/15/4	0.09	
BAL data				
BALF cell count (×10^3^/mL)	287 (130)	286 (128)	0.97	615 (572)
Neutrophil (%)	5.7 (9.5)	3.8 (4.7)	0.38	16.3 (29.5)
Lymphocyte (%)	13.5 (7.2)	11.7 (12.8)	0.63	31.5 (29.7)
Macrophage (%)	78.9 (14.8)	81.8 (15.2)	0.55	50.7 (33.7)
Eosinophil (%)	1.9 (2.9)	2.6 (3.5)	0.49	0.9 (1.9)
CD4/CD8 ratio	2.1 (1.0)	6.3 (20.3)	0.43	2.3 (2.3)
Other data				
pH	7.424 (0.024)	7.413 (0.024)	0.18	NA
PaCO_2_	39.7 (2.9)	40.0 (4.0)	0.78	NA
PaO_2_	86.8 (12.5)	83.5 (9.4)	0.35	NA
HCO_3_^−^	25.4 (1.8)	25.0 (1.8)	0.50	NA
6MWT distance (m)	443 (72)	458 (106)	0.63	NA
6MWT minimum SpO_2_ < 88% (yes/no)	5/10	15/14	0.34	NA

Abbreviations: *ACPA* Anti-cyclic citrullinated peptide antibody, *RA-ILD* Rheumatoid arthritis-associated interstitial lung disease, *BMI* Body mass index, *WBC* White blood cell, *LDH* Lactate dehydrogenase, *CRP* C-reactive protein, *KL-6* Krebs von den Lungen-6, *SP-D* Surfactant protein D, *FVC* Forced vital capacity, *FEV*_*1*_ Forced expiratory volume in 1 s, *DLCO* Diffusing capacity of the lung for carbon monoxide, *BALF* Bronchoalveolar lavage fluid, *6MWT* six-minute walking test

*P*-value was calculated for the comparison of the valuables between IPF patients with BALF ACPA proportion high and low

### AIQCT indices in the BALF of IPF patients with high/low ACPA proportions

The automatically quantified indices of each parenchymal pattern as well as the bronchi and vessels were compared between IPF patients with high and low ACPA proportions. There were no significant differences in the volumes or percentages of reticulation, honeycomb, fibrosis, or ILD lesions, as shown in Table 2.

**Table 2:** Volumes and percentages of parenchymal CT pattern lesions quantified by AI-based software

	Volume (mL)			Percentage		
	IPF High ACPA proportion (*n* = 15)	IPF Low ACPA proportion (*n* = 29)	*p* value	IPF High ACPA proportion (*n* = 15)	IPF Low ACPA proportion (*n* = 29)	*p* value
All	3658 (973)	3850 (1056)	0.58	NA	NA	NA
Normal	2774 (929)	2971 (998)	0.55	74.9 (8.9)	76.4 (10.9)	0.66
GGO	130 (63)	133 (115)	0.92	3.8 (2.2)	3.8 (3.3)	0.99
Reticular	211 (132)	203 (176)	0.89	6.4 (4.6)	5.4 (3.9)	0.48
Consolidation	16 (12)	20 (19)	0.45	0.4 (0.3)	0.6 (0.7)	0.48
Honeycomb	31 (47)	22 (35)	0.48	1.0 (1.6)	0.6 (0.7)	0.25
Nodule	27 (21)	23 (18)	0.57	0.8 (0.8)	0.6 (0.4)	0.24
Other	34 (18)	31 (14)	0.54	1.1 (0.7)	0.9 (0.4)	0.25
Hyperlucent	82 (198)	71 (142)	0.85	1.9 (3.9)	1.9 (3.9)	0.98
Bronchi	142 (51)	147 (55)	0.76	4.1 (1.7)	4.0 (1.5)	0.83
Vessels	212 (74)	228 (75)	0.52	5.7 (1.1)	6.0 (1.5)	0.56
Fibrosis	242 (154)	225 (208)	0.79	7.3 (5.4)	5.9 (4.5)	0.38
ILD	372 (173)	358 (285)	0.87	11.1 (6.5)	9.7 (6.1)	0.50

Abbreviations: *ACPA* Anti-cyclic citrullinated peptide antibody, *GGO* ground‒glass opacity, *ILD* interstitial lung disease

*P*-value was calculated for the comparison of the valuables between IPF patients with BALF ACPA proportion high and low

### Clinical impact of the proportion of ACPAs in the BALF on patient outcomes

The follow-up information after BALF collection is shown in Table 3. The median follow-up period was 128.4 months (range 4.1–161.0 months) in patients with IPF. No subjects in either group developed RA. There was no difference in treatment options after BALF collection. During the follow-up, 11 patients (73%) in the group with high ACPA proportions died, and 20 patients (69%) in the group with low ACPA proportions died. The causes of death are also shown in Table 3. Two out of the 11 (18%) patients in the group with high ACPA proportions died of acute exacerbation, whereas seven out of 20 (35%) patients in the group with low ACPA proportions died of acute exacerbation. No patients died of COVID-19.

**Table 3:** Follow-up information of IPF patients after BAL procedure

	IPF High ACPA proportion (*n* = 15)	IPF Low ACPA proportion (*n* = 29)
Treatment after BAL		
Antifibrotics	10	15
Immunosuppressant	1	2
Antifibrotics plus immunosuppressant	2	0
No treatment	2	12
Total death cases	11	20
Cause of death		
AE-ILD	2	7
Chronic respiratory failure	3	4
Lung cancer	2	1
Infectious pneumonia	2	2
Diseases in other systems	1	3
Unknown cause	1	3

Abbreviations: *ACPA* Anti-cyclic citrullinated peptide antibody, *BAL* Bronchoalveolar lavage, *AE-ILD* Acute exacerbation of interstitial lung disease

**Fig. 2:**
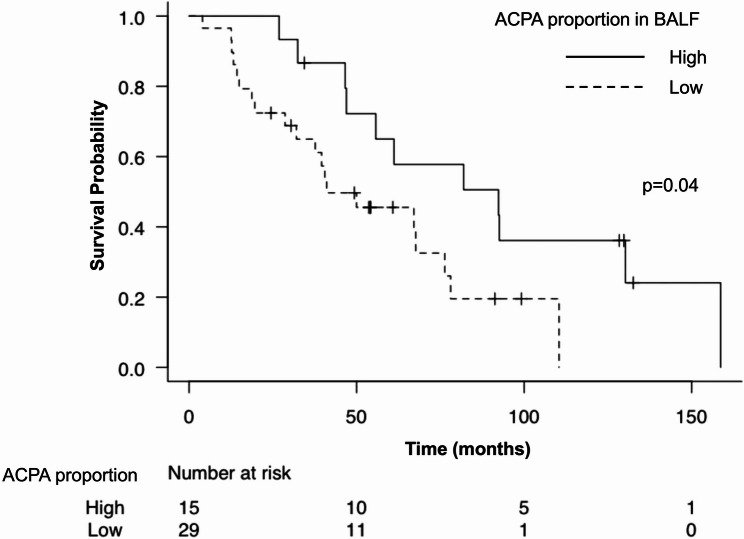
Survival of IPF patients stratified by BALF ACPA proportion. Kaplan‒Meier curves for patients with IPF stratified by the proportion of anti-cyclic citrullinated peptide antibody (ACPA) in bronchoalveolar lavage fluid (BALF) (high or low). *P*=0.04 according to the log rank test

A Cox proportional hazards model was used to perform the survival analysis. According to the univariate models, older age (HR 1.07, 95% CI 1.01–1.13, *p* = 0.03), a greater number of smoking pack-years (HR 1.01, 95% CI 1.00–1.02, *p* = 0.03), lower %FVC (HR 0.97, 95% CI 0.95–0.99, *p* < 0.01), lower %DLCO (HR 0.94, 95% CI 0.91–0.98, *p* < 0.01), higher GAP stage (stage II: HR 3.38, 95% CI 1.17–9.75, *p* = 0.02, stage III: HR 9.18, 95% CI 2.80–30.12, *p* < 0.01) were significantly associated with worse survival. Moreover, a high BALF ACPA proportion was significantly associated with better survival compared with a low ACPA proportion (HR 0.42, 95% CI 0.19–0.96, *p* = 0.04) (Table 4). The fractions of lymphocytes or neutrophils in the BALF did not predict survival (Table 4). The Kaplan‒Meier survival curve revealed that IPF patients with a high ACPA proportion had significantly better survival than those with a low ACPA proportion (median overall survival time: 92.4 months vs. 41.1 months, *p* = 0.04 according to the log rank test, Fig. 2).

**Table 4:** Univariate Cox proportional hazard models for all-cause mortality

	HR	95% CI	*p* value
Age	1.07	1.01–1.13	0.03
Male sex	2.44	0.82–7.26	0.11
BMI (kg/m^2^)	0.99	0.91–1.08	0.86
Pack-years	1.01	1.00–1.02	0.03
LDH (U/L)	1.00	0.99–1.01	0.51
KL-6 (U/mL)	1.00	0.99–1.00	0.32
%FVC	0.97	0.95–0.99	< 0.01
%DLCO	0.94	0.91–0.98	< 0.01
GAP stage I	Ref		
stage II	5.89	2.06–16.83	< 0.01
stage III	20.60	4.90–86.61	< 0.01
High BALF ACPA proportion (vs. low)	0.42	0.19–0.96	0.04
BALF cell count (×10^3^/mL)	1.00	0.99–1.00	0.32
BALF lymphocytes (%)	0.99	0.95–1.02	0.43
BALF neutrophils (%)	1.02	0.97–1.07	0.50

Abbreviations: *BMI* Body mass index, *LDH* Lactate dehydrogenase, *KL-6* Krebs von den Lungen-6, *FVC* Forced vital capacity, *DLCO* Diffusing capacity of the lung for carbon monoxide, *GAP* Gender-Age-Physiology, *BALF* Bronchoalveolar lavage fluid, *ACPA* Anti-cyclic citrullinated peptide antibody

### Comparison of clinical features and outcomes between BALF IgA-ACPA positive and negative patients with IPF

Additionally, IgA-ACPA was qualitatively measured in the BALF in 34 IPF patients due to the limited availability of archived BALF samples. Among these patients, 20 were positive and 14 patients were negative for BALF IgA ACPAs. Patients who were IgA ACPA-negative had a higher smoking exposure (pack-years) than those who were IgA ACPA-positive (*p* < 0.05); however, no significant differences were observed in other clinical features (Supplementary Table 1). Furthermore, there were no significant difference in survival between IgA ACPA- positive and -negative patients (Supplementary Fig. 1).

## Discussion

In the present study, the ACPA proportion (ACPA-IgG level adjusted by the total IgG level) in the BALF was investigated in patients with IPF. First, the proportion of ACPAs in the BALF was significantly lower in patients with IPF than in those with RA-ILD. Second, the IPF group with high ACPA proportions was younger and had more females. Importantly, patients with a high ACPA proportion had significantly better outcomes than those with a low ACPA proportion. To our knowledge, this is the first study to investigate the impact of ACPA levels in the BALF on the outcomes of patients with IPF.

The present study revealed better outcomes in IPF patients with a high ACPA proportion than in those with a low ACPA proportion, although there was no significant difference in the PFT indices, such as the %FVC, %DLCO, and GAP stage, between these two groups. Notably, the serum ACPA levels of all but one patient with IPF were negative. These results suggest that, rather than the serum ACPA level, the BALF ACPA proportion might be an important biomarker for predicting survival independent of disease severity. Previous studies showed that cigarette smoking may promote protein citrullination in the lung through local activation of peptidylarginine deiminase enzymes and lead to ACPA formation and the development of RA-ILD [21, 31–36]. Therefore, the lung may serve as a primary site of ACPA production in some patients with RA-ILD. We speculated that IPF phenotype with a high ACPA proportion might share pathogenic mechanisms with RA-ILD, including active ACPA production in the lung driven by upregulation of the local citrullination pathway and have favourable outcomes. In addition, patients with a high ACPA proportion in our cohort were significantly younger and more likely to be female than those with a low ACPA proportion were. Although these characteristics differ from the typical IPF profile, they partially overlap with the features reported for RA-ILD, suggesting that elevated local ACPA may reflect an immune pathway shared with autoimmune-related ILD and indicate a distinct IPF phenotype.

It remains unclear whether there is a link between serum ACPA positivity and high ACPA proportion in BALF. Zheng et al. compared the outcomes between seropositive (serum rheumatoid factor positive or ACPA positive) and seronegative non-connective tissue disease-associated ILD and found that seropositivity was not associated with improved outcomes [37]. Based on our results, the BALF ACPA proportion may provide more detailed information regarding clinical features and outcomes than serum ACPA levels. Reynisdottir et al. reported that parenchymal CT abnormalities such as emphysema, fibrosis, and ground glass opacities, as well as increased expression of citrullinated proteins in bronchial tissue, were observed in serum ACPA-positive patients with early RA. They further demonstrated ACPA titers were relatively higher in the BALF than in paired serum samples obtained from the same patients [36]. Together with other reports [38, 39], these findings suggest that the local ACPA production in the lung may be an initial event preceding systemic autoimmunity and the progression of ILD. However, it remains uncertain whether an increased ACPA proportion in BALF precedes serum ACPA positivity in some patients with IPF, as serum ACPA was measured only at the time of diagnosis in our study. Therefore, prospective long-term follow-up studies are required to clarify the temporal relationship between BALF and serum ACPA levels and their clinical consequences.

Recent studies have identified the presence of several autoantibodies in a subset of patients with IPF that are not routinely measured in clinical practice [40–44], and notably, some autoantibodies have been associated with disease progression or a worse prognosis [41, 42]. These reports suggest that autoimmunity may play a role in the pathogenesis of IPF. The present study revealed that a substantial number of IPF patients have increased ACPA levels in the BALF, although the serum ACPA level is negative, suggesting that some patients with IPF might develop pulmonary fibrosis due to local antibody-mediated tissue damage, not a systemic autoimmune reaction. A recent study by Boustani et al. supports our hypothesis [45]. The IPF phenotype characterized by localized ACPA-mediated pulmonary fibrosis might be less progressive than the other phenotypes.

Whether RA-ILD patients with a UIP pattern (RA-UIP) have better outcomes than IPF patients is controversial. Song et al. reported that 57 patients with RA-UIP (30 with RA-UIP confirmed by surgical lung biopsy) had better prognoses than did those with IPF [46], whereas Moua et al. reported that 24 patients with biopsy-confirmed RA-UIP had prognoses similar to those of patients with IPF [47]. Another study by Solomon et al. compared the outcomes of RA-UIP with those of RA-ILD with nonspecific interstitial pneumonia classified by CT patterns and reported that the median survival time of RA-UIP patients is greater than 10 years, which is much better than that of IPF patients [1, 2]. In this study, the median survival time of IPF patients with a high ACPA proportion was 92.4 months, which is apparently longer than that reported previously for IPF patients.

Notably, there was no significant difference in the proportion of lymphocytes in the BALF between the group with high ACPA proportions and that with low ACPA proportions, and the proportion of lymphocytes in the BALF was not significantly associated with survival in the present study. Although several studies with small sample sizes have reported increased proportions of lymphocytes in the BALF of RA-ILD patients [48, 49], the clinical utility of the proportion of lymphocytes in the BALF of RA-ILD patients remains unknown [50]. Furthermore, another study revealed that there was no difference in the proportion of lymphocytes in the BALF between IPF patients and RA-UIP patients [31]. In the present study, the lymphocyte count in BALF was significantly correlated with the total IgG level (ρ = 0.36, *p* = 0.02) but not with the ACPA proportion in BALF (ρ = 0.15, *p* = 0.34), suggesting that BALF lymphocytes may reflect general adaptive immune activity but not specific immune processes such as activation of the citrullination pathway. Therefore, it might be useful to measure not only cell counts but also ACPA levels in BALF to detect specific phenotypes with better outcomes.

In this study, the ACPA proportion was measured in the BALF of RA-ILD patients, and although the average ACPA proportion was greater in RA-ILD patients than in IPF patients, the ACPA proportion was not high in all patients with RA-ILD. One of the possible reasons for this result is that multiple factors can contribute to the development of RA-ILD. Although the lung might be the initial and important site for ACPA production in some RA-ILD patients [21, 31–36], other environmental, infectious, or genetic factors may also be involved in the development of RA and RA-ILD [21, 33, 34].

In the present study, we also quantified CT features via AI-based software that we recently developed [30], and there was no significant difference in the indices of CT features between IPF patients with high and low ACPA proportions. Although the extent of fibrosis can be an important biomarker for predicting outcomes in IPF patients, the ACPA proportion might help to identify IPF phenotypes with favourable outcomes that CT features are not able to detect.

There are several limitations in this study. First, the number of subjects was small, which may have limited the statistical power, particularly for subgroup analyses. Therefore, the possibility of type II error cannot be excluded, and the observed effect sizes may be unstable. Nevertheless, the associations observed were directionally consistent and biologically plausible, supporting the relevance of local ACPA in the lung. Given the exploratory nature of this study, the findings should be interpreted with caution and require validation in larger, independent cohorts. Next, the recovery rate after BALF collection varies depending on the patient. To address this issue, we adjusted the ACPA level by the total IgG level as described in a previous study [36]. Moreover, this study did not evaluate serum IgA ACPA levels. Although IgA ACPAs in the BALF were qualitatively assessed in a subset of patients, the retrospective nature of the study and the limited availability of archived BALF samples, together with the use of a qualitative assay, precluded robust quantitative or prognostic analyses. Therefore, our findings regarding IgA ACPA in the BALF should be interpreted with caution. Although we focused on IgG ACPA because this subtype has been linked to autoimmune activity relevant to IPF, IgA ACPA may provide additional, complementary insight. Given that IgA often predominates at mucosal surfaces and may reflect airway mucosal immune activation, future studies assessing serum and BALF IgA ACPA are important to determine whether IgA-mediated pathways contribute independently to IPF pathogenesis and prognosis. In addition, exploring other autoantibodies or immune markers in the BALF or lung tissue may provide a more comprehensive understanding of local autoimmune activity and its relevance to IPF prognosis.

## Conclusion

Compared with IPF patients with low ACPA proportions, IPF patients with high ACPA proportions were younger, more likely to be female, and had better survival. The corrected ACPA level might be an important biomarker for identifying phenotypes associated with favourable outcomes in IPF patients.

## Supplementary Information

Supplementary Material 1.

## Data Availability

The data that support the findings of this study are not publicly available due to privacy or ethical restrictions and are available from the corresponding author upon a reasonable request.

## Abbreviations

IPF: Idiopathic Pulmonary Fibrosis
RA: Rheumatoid Arthritis
ILD: Interstitial Lung Disease
UIP: Usual Interstitial Pneumonia
HRCT: High Resolution Computed Tomography
ACPA: Anti-cyclic citrullinated peptide antibody
BALF: Bronchoalveolar lavage fluid
AIQCT: Artificial intelligence-based quantitative CT image analysis software
