# Supplementary material for: Anti-cyclic citrullinated peptide antibody in bronchoalveolar lavage fluid of patients with idiopathic pulmonary fibrosis
Source: BMC Pulm Med. 2026 Jan 8;26:54. doi: 10.1186/s12890-025-04088-9 (PMC12871011; doi:10.1186/s12890-025-04088-9)
Supplement: Supplementary file 1 — Supplementary Material 1. [file 12890_2025_4088_MOESM1_ESM.docx]

**Supplementary Table 1. Characteristics of IPF patients with positive and negative BALF IgA ACPA**

|  | IPF  IgA ACPA positive  (n=20) | IPF  IgA ACPA negative  (n=14) | *p* value |
| --- | --- | --- | --- |
| **Demographic data** |  |  |  |
| Age | 71 (9) | 67 (9) | 0.26 |
| Sex M/F | 15 / 5 | 13 / 1 | 0.36 |
| BMI (kg/m^2^) | 25.2 (4.3) | 25.7 (3.7) | 0.75 |
| Smoking status  Current / Past / Never | 0 / 17 / 3 | 1 / 12 / 1 | 0.58 |
| Pack-years | 33.6 (25.5) | 58.2 (44.4) | 0.049 |
| WBC (/mm^3^) | 7014 (1774) | 7045 (925) | 0.95 |
| LDH (U/L) | 223 (44) | 226 (28) | 0.85 |
| CRP (mg/dL) | 0.2 (0.3) | 0.2 (0.1) | 0.92 |
| KL-6 (U/mL) | 967 (599) | 989 (592) | 0.92 |
| SP-D (ng/mL) | 245 (132) | 339 (351) | 0.28 |
| Serum ACPA +/– | 0 / 20 | 0 / 14 | 1.00 |
| **Pulmonary function testing** |  |  |  |
| %FVC | 76.5 (20.9) | 82.4 (20.7) | 0.42 |
| FEV_1_/FVC | 85.7 (6.6) | 83.9 (8.5) | 0.50 |
| %DLCO | 54.0 (19.9) | 52.3 (12.7) | 0.78 |
| **GAP stage (I/II/III)** | 10 / 7 / 3 | 5 / 9 / 0 | 0.17 |
| **BAL data** |  |  |  |
| BALF cell count (×10^3^/mL) | 278 (98) | 288 (163) | 0.83 |
| Neutrophil (%) | 4.0 (5.7) | 4.4 (8.2) | 0.87 |
| Lymphocyte (%) | 9.5 (8.1) | 12.8 (11.7) | 0.34 |
| Macrophage (%) | 84.0 (12.3) | 80.5 (17.1) | 0.49 |
| Eosinophil (%) | 2.6 (3.0) | 2.4 (4.0) | 0.87 |
| CD4/CD8 ratio | 3.3 (3.5) | 2.4 (1.3) | 0.37 |
| **Other data** |  |  |  |
| pH | 7.42 (0.03) | 7.42 (0.03) | 0.34 |
| PaCO_2_ | 40.3 (4.0) | 39.1 (3.8) | 0.37 |
| PaO_2_ | 85.8 (10.9) | 82.3 (11.0) | 0.37 |
| HCO_3_^-^ | 25.3 (2.2) | 24.9 (1.5) | 0.54 |
| 6MWT distance (m) | 438 (111) | 468 (95) | 0.42 |
| 6MWT minimum SpO_2_ <88% (yes/no) | 10 / 10 | 7 / 7 | 1.00 |

Abbreviations: *ACPA* anti-cyclic citrullinated peptide antibody; *RA-ILD* rheumatoid arthritis-associated interstitial lung disease; *BMI* body mass index; *WBC* white blood cell; *LDH* lactate dehydrogenase; *CRP* C-reactive protein; *KL-6* Krebs von den Lungen-6; *SP-D* surfactant protein D; *BALF* bronchoalveolar lavage fluid; *FVC* forced vital capacity; *FEV_1_* forced expiratory volume in 1 second; *DLCO* diffusing capacity of the lung for carbon monoxide; *6MWT* six-minute walking test.

**Supplementary Figure 1.**


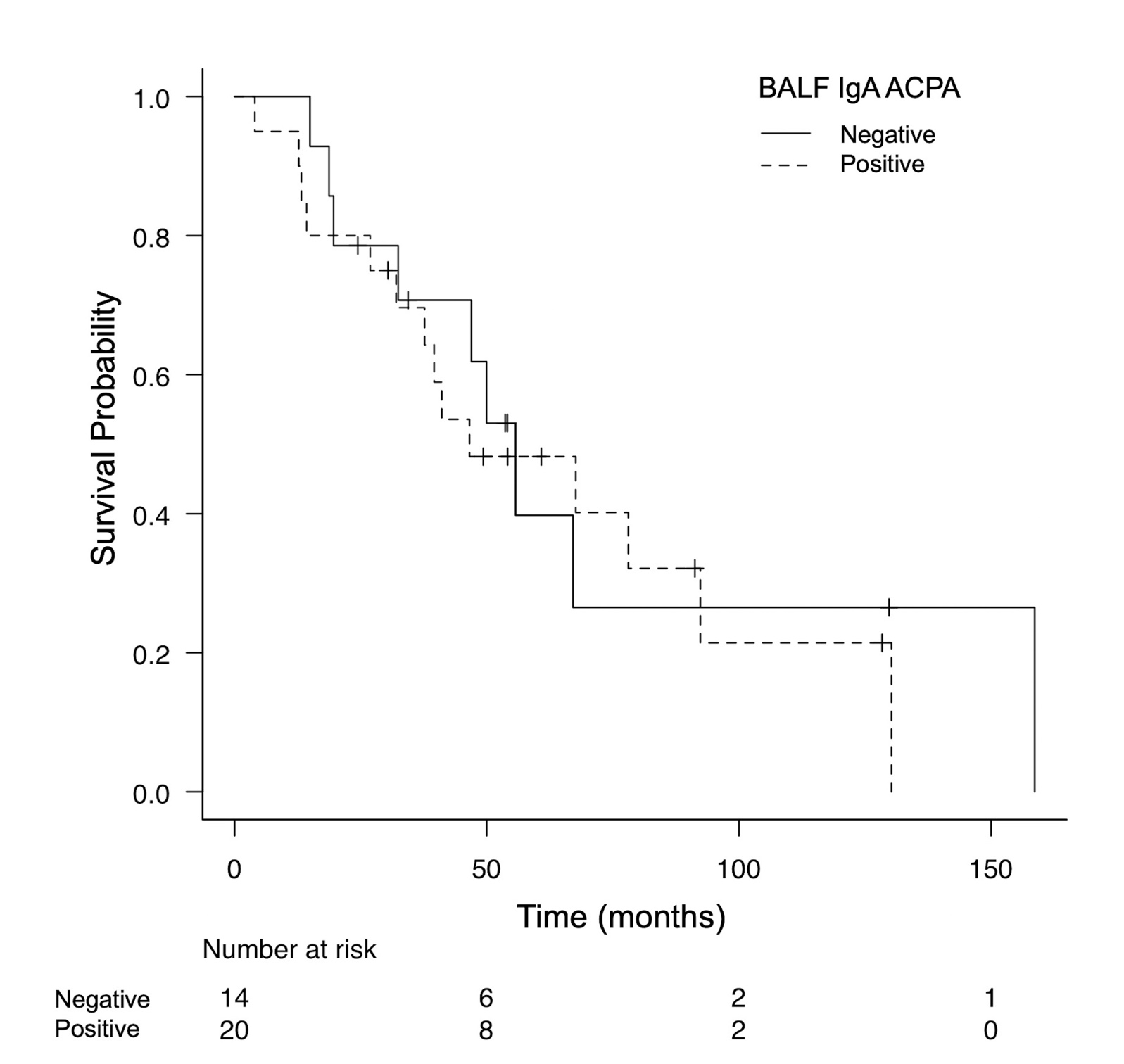


**Supplementary Figure 1.** Kaplan-Meier curves for IPF patients with positive and negative BALF IgA ACPA. Median survival did not differ between BALF IgA ACPA-positive and -negative patients. (log-rank p=0.66).
